# Real‐world etiologies and treatments of pediatric short bowel syndrome in Japan

**DOI:** 10.1111/ped.15258

**Published:** 2022-09-26

**Authors:** Yuko Tazuke, Eri Udagawa, Tsunekazu Mizushima, Shiro Nakamura, Jovelle Fernandez, Hiroomi Okuyama

**Affiliations:** ^1^ Department of Pediatric Surgery Osaka University Graduate School of Medicine Osaka Japan; ^2^ Japan Medical Office Takeda Pharmaceutical Company Limited Tokyo Japan; ^3^ Department of Therapeutics for Inflammatory Bowel Diseases Osaka University Graduate School of Medicine Osaka Japan; ^4^ 2nd Department of Internal Medicine Osaka Medical and Pharmaceutical University Osaka Japan

**Keywords:** child, etiology, parenteral nutrition, short bowel syndrome, weaning

## Abstract

**Background:**

Short bowel syndrome (SBS) is a rare disease that can result in intestinal failure (IF). Short bowel syndrome intestinal failure leads to stunted growth and development and high mortality rates. The primary goal of treatment is to enhance intestinal adaptation and nutrient absorption. Parenteral nutrition (PN) is used to support this process until enteral autonomy can be restored. Some patients experience prolonged partial or complete dependency on PN and face an increased risk of life‐threatening catheter‐related bloodstream infections and intestinal failure‐associated liver disease. This study aimed to provide real‐world insights into the patient characteristics and treatment dynamics of PN‐dependent children with SBS‐IF in Japan.

**Methods:**

This retrospective observational study used anonymized information from a large hospital‐based medical insurance database to identify pediatric patients who received PN for ≥6 months between April 2008 and January 2020. The primary endpoint was weaning from PN. Secondary endpoints included duration and complications of PN.

**Results:**

Forty‐eight children (mean age, 2.9 years) were eligible for inclusion. The most common causes of SBS‐IF were mechanical bowel obstruction, functional bowel disorders, and Hirschsprung's disease. Twenty‐two patients (45.8%) were weaned from PN during the study. The mean time to first weaning was 464.2 days and five patients (22.7%) restarted PN. The mean total duration of PN was 692.6 days in weaned patients and 1,170.9 days in unweaned patients. The most frequent complications were sepsis, catheter infections (both 79.2%), and liver dysfunction (64.6%).

**Conclusions:**

Pediatric patients with SBS‐IF faced difficulties when weaning off PN and rates of life‐threatening complications were high.

Short bowel syndrome (SBS) is a malabsorptive state resulting from physical or functional loss of a portion of the small intestine that can result in intestinal failure (IF).^1^, ^2^, ^3^, ^4^ Short bowel syndrome IF occurs comparatively rarely in children but is associated with high mortality rates, especially in newborns.^5^

Patients with SBS‐IF require long‐term nutritional support to prevent malnutrition and for growth; hence the primary treatment goals are to enhance intestinal adaptation and restore enteral autonomy.^6^ In the acute phase (first 3–4 weeks) this takes the form of parenteral nutrition (PN).^3^ Parenteral nutrition should be tailored to the patient and include adequate amino acids, lipids, carbohydrates, micronutrients, and electrolytes to sustain development and growth.^3^, ^4^, ^7^ Ideally, during the first few weeks after initiating management strategies, spontaneous structural and functional changes in the remaining small bowel should result in gradual improvements in nutrient absorption. This allows weaning from PN and gradual transition to enteral nutrition and, eventually, oral nutrition.^2^ However, some cases are not clinically successful and require longer term partial or complete PN dependency.^8^ This increases the risk of complications, such as catheter‐related bloodstream infections (CRBSI) and intestinal failure‐associated liver disease (IFALD).^5^

Based on Japanese Ministry of Health, Labor and Welfare data,^9^ we estimate that there are about 1,000 patients (adults and children) with SBS who are certified for disability owing to IF. To date, perspectives on the causes of SBS in Japan have been derived from clinical case reports, which may not provide a complete picture of the etiology of SBS in pediatric and adult patients throughout the country. Specifically, existing studies have narrow scopes and focus on nutritional impact and surgical procedures, giving limited information on the outcomes of different treatments for SBS in the Japanese population.^10^, ^11^

This study used electronic health records to identify the causes of SBS, patterns of treatment, and subsequent outcomes, in PN‐dependent Japanese children <16 years.

## Methods

### Data source

This retrospective observational study used anonymized electronic hospital‐based health‐insurance claims and Diagnosis Procedure Combination data, provided by Medical Data Vision Co. Ltd. (MDV, Tokyo, Japan). This large‐scale, acute‐care hospital‐based database covers inpatient and outpatient data from >399 hospitals (of a total of 8,300 in Japan) and 30.15 million patients (of a total population of 126 million). Secondary analysis of such a database is reasonable for the purpose of this study because its size allows rare diseases to be captured and most small bowel resections leading to SBS are performed in the hospitals that are covered.

The MDV database fulfills the anonymity requirements of the revised Personal Information Protection Law of Japan. It includes the following information: disease names coded using the World Health Organization's International Statistical Classification of Diseases and Related Health Problems 10th Revision (ICD‐10) coding scheme; disease names coded using Japanese Disease Name Codes for health‐insurance reimbursement; medical procedures coded using Japanese Procedure Codes; and prescription information, including generic drug names. In the Japanese claim database, diagnosis of SBS is determined by a disease receipt code of 8841646, so the ICD‐10 code cannot be used for only SBS. Data collected from 1 April 2008 to 31 January 2020 were analyzed to investigate patients <16 years who received PN for ≥6 months (183 days).

The study was conducted according to the Declaration of Helsinki and the International Society for Pharmacoepidemiology Guidelines for Good Pharmacoepidemiology Practices.^12^ It was carried out retrospectively using anonymized database data, following the Ethical Guidelines for Medical and Health Research Involving Human Subjects, issued by the Japanese Ministry of Health, Labor, and Welfare.^13^ As investigators could only access anonymized information, in accordance with the aforementioned ethical guidelines, institutional ethics approval and informed consent were not required.

### Definitions

**Fig. 1:**
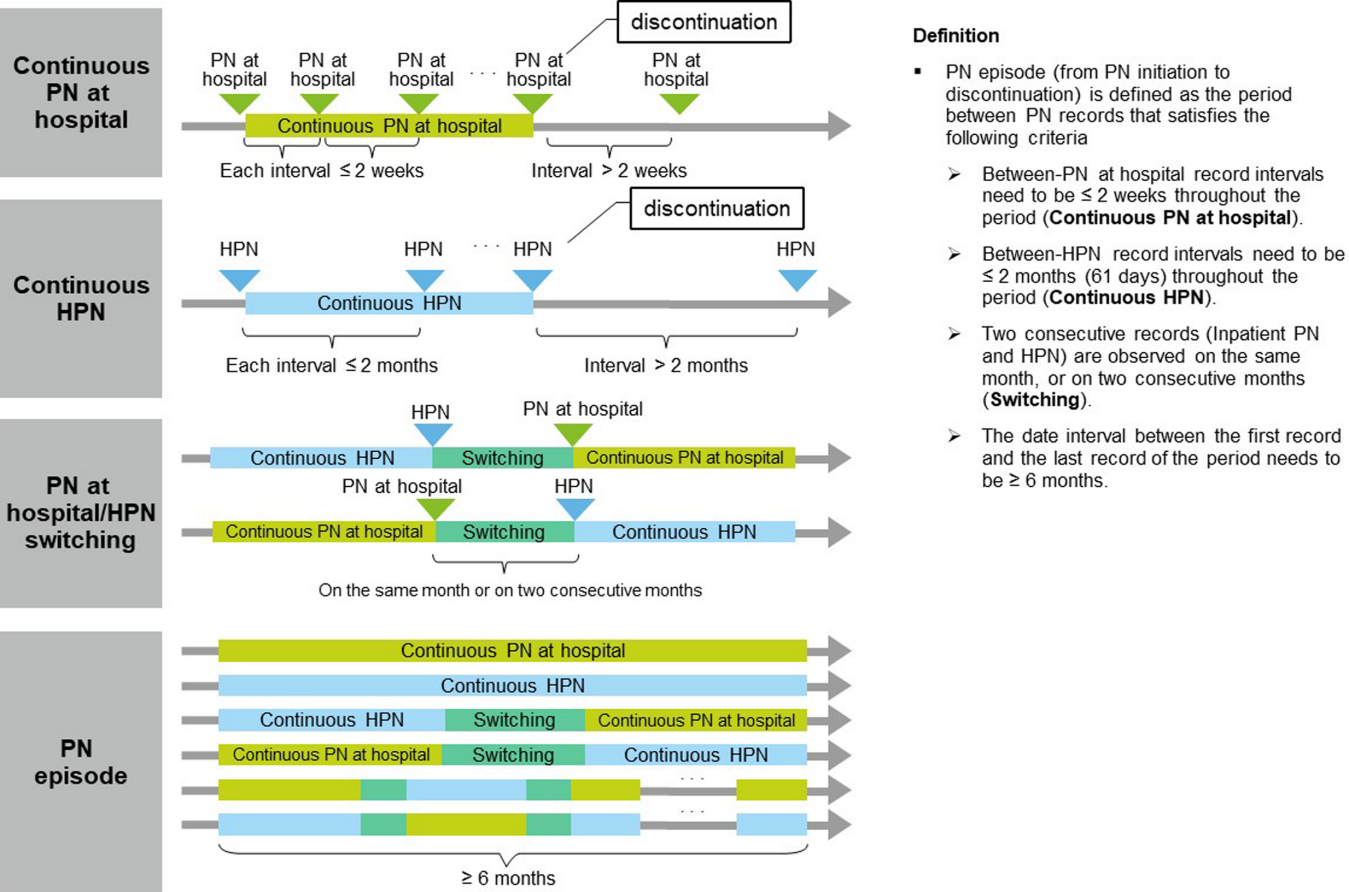
Schematic diagram representing definitions of parenteral nutrition episodes. Switching between PN at hospital and HPN was treated as a PN episode. HPN, home parenteral nutrition; PN, parenteral nutrition.

Parenteral nutrition was defined as all PN and home parenteral nutrition (HPN) was defined as PN at home. Parenteral nutrition at the hospital included inpatients and outpatients. A PN episode was defined as PN administration recorded continuously over ≥6 months; the index PN event was defined as the first episode recorded during the study period and the index date was the date of initiation of the index event (Fig. 1).

Eligible patients received PN at the hospital (continuous PN at hospital treatment records with intervals of ≤2 weeks between PN records throughout the period) or HPN – continuous PN with intervals of ≤2 months (61 days) between HPN treatment records. Patients switching between PN at the hospital and HPN management should have had two consecutive PN treatment records in the same month, or 2 consecutive months.

The 365 days prior to the index date are defined as the “look‐back period” and the period after the index date as the “follow‐up period.” The duration of these periods was not predetermined: patients were followed for as long as they could be tracked using the same identification number. If the patient's first record was created on the index date (most likely because of an emergency event), the look‐back period is defined as the index date.

### Selection of study cohort

Patients' medical and surgical histories during the look‐back period were recorded. Short bowel syndrome‐related surgery included resection of small‐bowel tumors and diverticula, resection of small bowel obstruction, surgery for intestinal malrotation, colostomy, enterostomy, resection of bowel obstruction (total or partial resection; includes resection of tumors), closure of fistulae in the small intestine (with resection of the intestinal tract), and colectomy (small incision). Children who were eligible for inclusion in this analysis were aged between 0 days (newborn) and <16 years as of the index date. They were required to have had at least one PN episode during the study period and a clinical history including one of the following: a record of SBS during the look‐back period and/or the follow‐up period or at least one disease known to cause SBS in the look‐back period and surgery associated with SBS (e.g., any small intestine restriction surgeries during the look‐back period or intestinal lengthening surgery during either the look‐back or the follow‐up period). There were no study exclusion criteria.

### Endpoints

The primary endpoint was weaning from PN in patients who had continued PN for ≥6 months. Successful weaning was defined by intervals of >2 months between PN records or intervals of >2 months (61 days) between the date of the last PN record and the last date of the follow‐up period.

Secondary endpoints were transfer to HPN, complications related to PN, PN restart, PN duration (index and total), nature of PN injections, comedications, requirements for nutritional and nursing care support, SBS‐related surgery, number and duration of hospitalizations, activity of daily living (ADL) scores (for eating, transfer, personal hygiene and grooming, toilet use, bathing, walking, stairs, clothing, feces management, and urination management) at discharge, and destination after discharge. Further analyses were carried out for patients who died during the study period.

### Statistical analysis

This is a descriptive study, so no tests were conducted to estimate statistical significance. Continuous variables were reported using means (standard deviation, SD) or medians (interquartile range, IQR) and categorical variables were reported using frequencies and percentages. Results were further stratified by age, weaning (or not) from PN, circumstances of transfer to HPN, and requirement for high‐calorie PN. All statistical analyses were conducted using SAS version 9.4 (SAS Institute; Tokyo, Japan); missing/incomplete data were not imputed.

## Results

### Patient characteristics

**Fig. 2:**
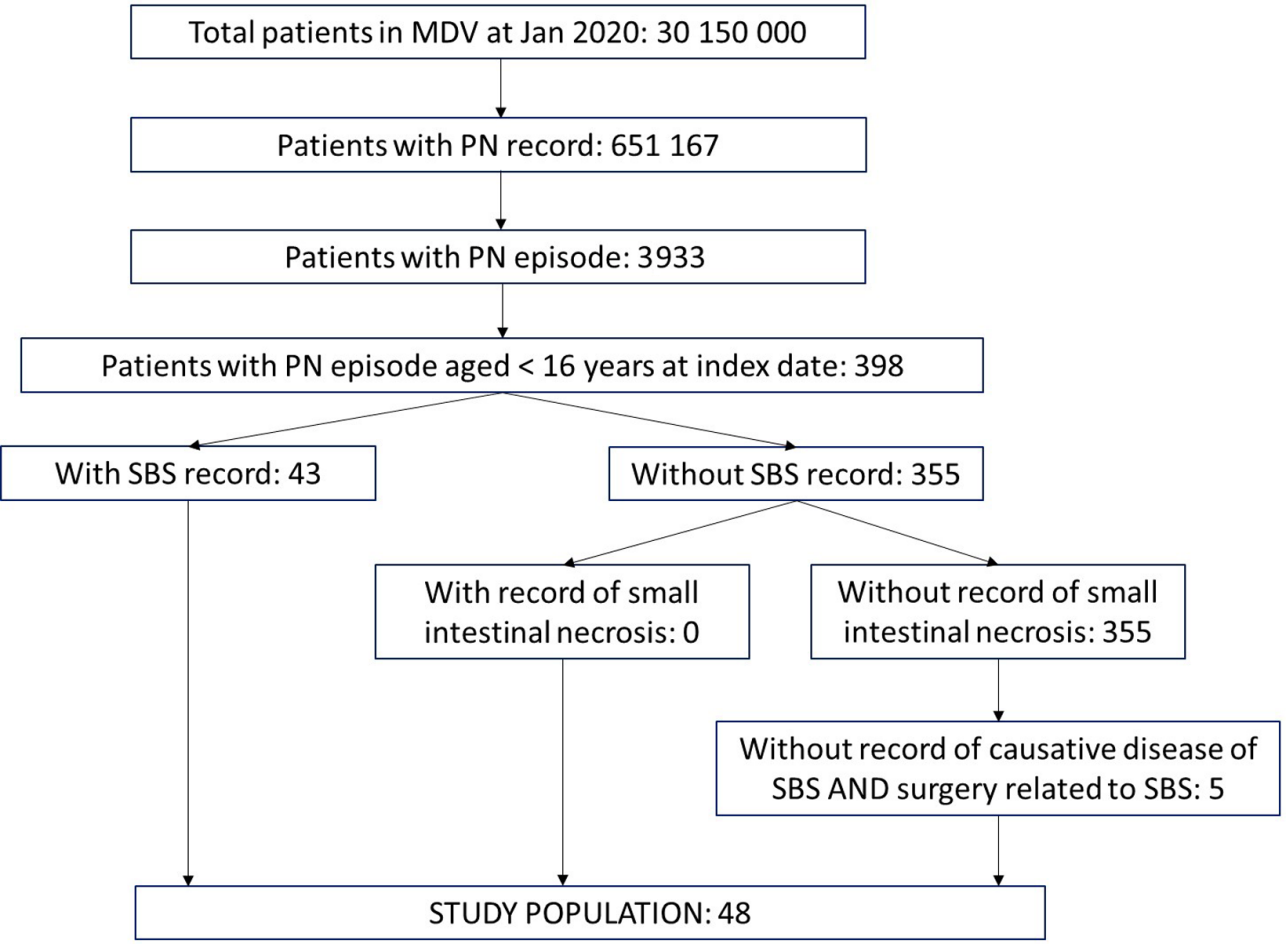
Patient disposition. MDV, Medical Data Vision; PN, parenteral nutrition; SBS, short bowel syndrome.

Between 1 April 2008 and 31 January 2020, we identified 48 pediatric patients who were eligible for inclusion in the study (Fig. 2). There were equal numbers of male and female patients and their mean age was 2.9 ± 3.7 years at the index date (Table 1).

**Table 1:** Patient demographics and characteristics

	*N* = 48
Male patients, *n* (%)	24 (50.0)
Mean age at index date, years (SD)	2.9 (3.7)
Age group, *n* (%)	
Infant (< 1 year)	22 (45.8)
Young child (1–5 years)	15 (31.3)
Child (6–15 years)	11 (22.9)
Difference from the standard height in different age groups, cm (SD)^†^	
Infant (<1 year)	−6.9 (5.0)
Young child (1–5 years)	−3.3 (3.9)
Child (6–15 years)	−3.5 (5.6)
Difference from the standard bodyweight in different age groups, kg (SD)^†^	
Infant (<1 year)	−4.9 (2.6)
Young child (1–5 years)	−1.9 (2.5)
Child (6–15 years)	−1.6 (2.4)
Most frequent^‡^ causes of SBS, *n* (%)	
Mechanical bowel obstruction	12 (25.0)
Functional bowel disorders	10 (20.8)
Hirschsprung's disease	10 (20.8)
Surgery for small bowel obstruction	8 (16.7)
Most frequent^‡^ comorbidities, *n* (%)	
Liver disease	23 (47.9)
Malnutrition	12 (25.0)
Dehydration	7 (14.6)
Type of index PN therapy, *n* (%)	
PN at hospital	32 (66.7)
Home PN	16 (33.3)

^†^ Measured only for children receiving PN at hospital.

^‡^ Top three medical causes of SBS; other medical causes accounted for 12.8% of cases.

PN, parenteral nutrition; SBS, short bowel syndrome; SD, standard deviation.

In more than half of the cases (53.3%), the index event started in a pediatric surgical unit. Most of the other cases started in pediatric (26.7%) or neonatal (16.7%) units. Nine out of thirty patients who were hospitalized (30.0%) were transferred from other hospitals. Two‐thirds of patients (32/48), with a mean age 1.5 ± 2.9 years, were receiving PN at hospital at the index date. The most frequent diseases leading to SBS were mechanical bowel obstruction (25.0%), functional bowel disorders, and Hirschsprung's disease (both 20.8%) (Table 2). The most common surgery was for the obstruction of the small bowel. Liver disease (present in 47.9% of patients) was the most frequent comorbidity identified in the look‐back period followed by malnutrition (25.0%).

**Table 2:** Full list of short bowel syndrome causative diseases

Category	Injury/illness
Gastroschisis	Abdominal wall rupture Omphalocele
Intestinal atresia (intestinal obstruction)	Neonatal intestinal obstruction Jejunal closure Ileal closure Small intestinal closure Generalized purulent peritonitis Perforated peritonitis Acute generalized peritonitis Peritonitis Meconium peritonitis Neonatal peritonitis
Necrotizing enterocolitis	Neonatal necrotizing enterocolitis Necrotizing enterocolitis Small intestinal necrosis Intestinal necrosis Ileal necrosis Meconium plug syndrome Perinatal intestinal perforation Perforated peritonitis Meconium peritonitis Neonatal peritonitis
Mechanical bowel obstruction	Meconium ileus Mesenteric hernia Internal hernia Torsion of the small intestine Strangulation ileus Adhesive ileus Obstructive ileus Postoperative adhesive ileus Total mesenteric disease Midgut volvulus
Functional bowel disorders	Paralytic ileus Megacolon Intestinal dysfunction Gastrointestinal disorders Hirschsprung's disease‐related diseases Chronic idiopathic pseudointestinal obstruction False ileus
Hirschsprung's disease	Whole colon Hirschsprung's disease Small intestinal Hirschsprung's disease Hirschsprung's disease
Mesenteric thrombosis/impaired blood flow	Acute superior mesenteric artery occlusion Thromboembolism

### Primary endpoint

**Fig. 3:**
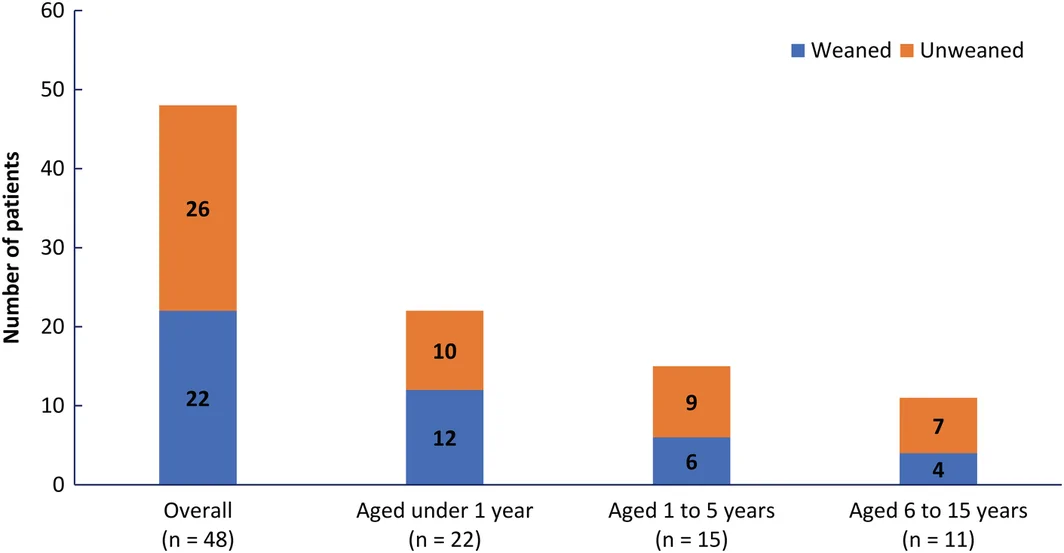
Effect of age on weaning rates from parenteral nutrition.

In total, 22 (45.8%) patients were weaned from PN. Weaning rates were highest for those aged under 1 year (12/22; 54.5%) and lowest for those aged 6 years and over (4/11; 36.4%) (Fig. 3). Compared with unweaned patients, lower proportions of weaned patients had liver disease (14/26; 53.8% vs. 9/22; 40.9%) and malnutrition (7/26; 26.9% vs. 5/22; 22.7%).

### Secondary endpoints

#### Duration of parenteral nutrition

The mean time from index date to first weaning was 464.2 ± 261.8 days. The mean duration of the index PN event was 424.6 ± 249.8 days in weaned patients and 1,026.0 ± 818.4 days in unweaned patients. Five patients (22.7%) who were weaned were subsequently restarted on PN; the total duration of PN was 692.6 days in weaned patients and 1,170.9 days in unweaned patients.

#### Delivery of parenteral nutrition

The majority of the patients (41/48; 85.4%) required high‐calorie infusions. The proportions of patients with liver disease (46.3% vs. 57.1%) and malnutrition (24.4% vs. 26.6%) who were or were not prescribed high‐calorie PN (usually delivering around 100 calories/kg/day for children) were similar. All patients with dehydration, chronic kidney disease, or diabetes were prescribed high‐calorie PN. During the index PN episode, all patients received anticoagulants and almost all (97.9%) also received antibiotics (Table 3). Just over 10% of patients received in‐hospital support from the nutritional support team. No patients receiving HPN required support from the nutritional support team.

**Table 3:** Complications of parenteral nutrition and comedications during the study period^†^ (*N* = 48)

Endpoint	Number of patients, *n* (%)
Complications of parenteral nutrition	
Catheter infection	38 (79.2)
Sepsis	38 (79.2)
Liver disease	31 (64.6)
Bacteremia	21 (43.8)
Mechanical complications	10 (20.8)
Venous thromboembolism	7 (14.6)
Comedications	
Anticoagulants	48 (100)
Antibiotics	47 (97.9)
Antidiarrheals	43 (89.6)
Gastric antisecretory agents	33 (68.8)

^†^ Observed in ≥10% of patients in both cases.

#### Complications of parenteral nutrition

**Fig. 4:**
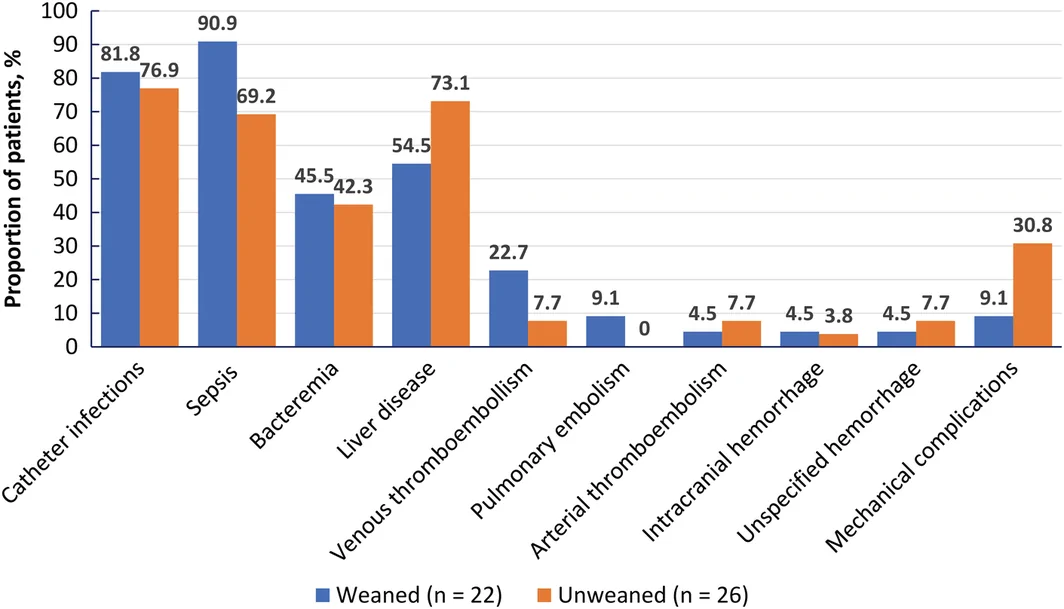
Frequencies of complications related to parenteral nutrition stratified by weaning status.

The most common complications of PN were CRBSIs (79.2%), sepsis (79.2%), and IFALD (64.6%; Table 3). When stratified, a greater proportion of patients in the weaned group developed sepsis (90.9% vs. 69.2% in patients who were not weaned); however, a lower proportion of patients in the weaned group developed IFALD (54.5% vs. 73.1%) and mechanical complications (e.g., bleeding, pneumothorax, nerve injury, or malignant arrhythmia) with their central venous catheters (9.1% vs. 30.8%; Fig. 4). Patients with CRBSIs (82.9% vs. 57.1%), sepsis (82.9% vs. 57.1%), and bacteremia (46.3% vs. 28.6%) were more likely to be prescribed high‐calorie PN than not. Conversely, patients with venous thromboembolism (42.9% vs. 9.8%), pulmonary embolism (14.3% vs. 2.4%) and unspecified hemorrhage (14.3% vs. 4.9%) were less likely to be prescribed high‐calorie PN.

#### Transfer to home parenteral nutrition

**Fig. 5:**
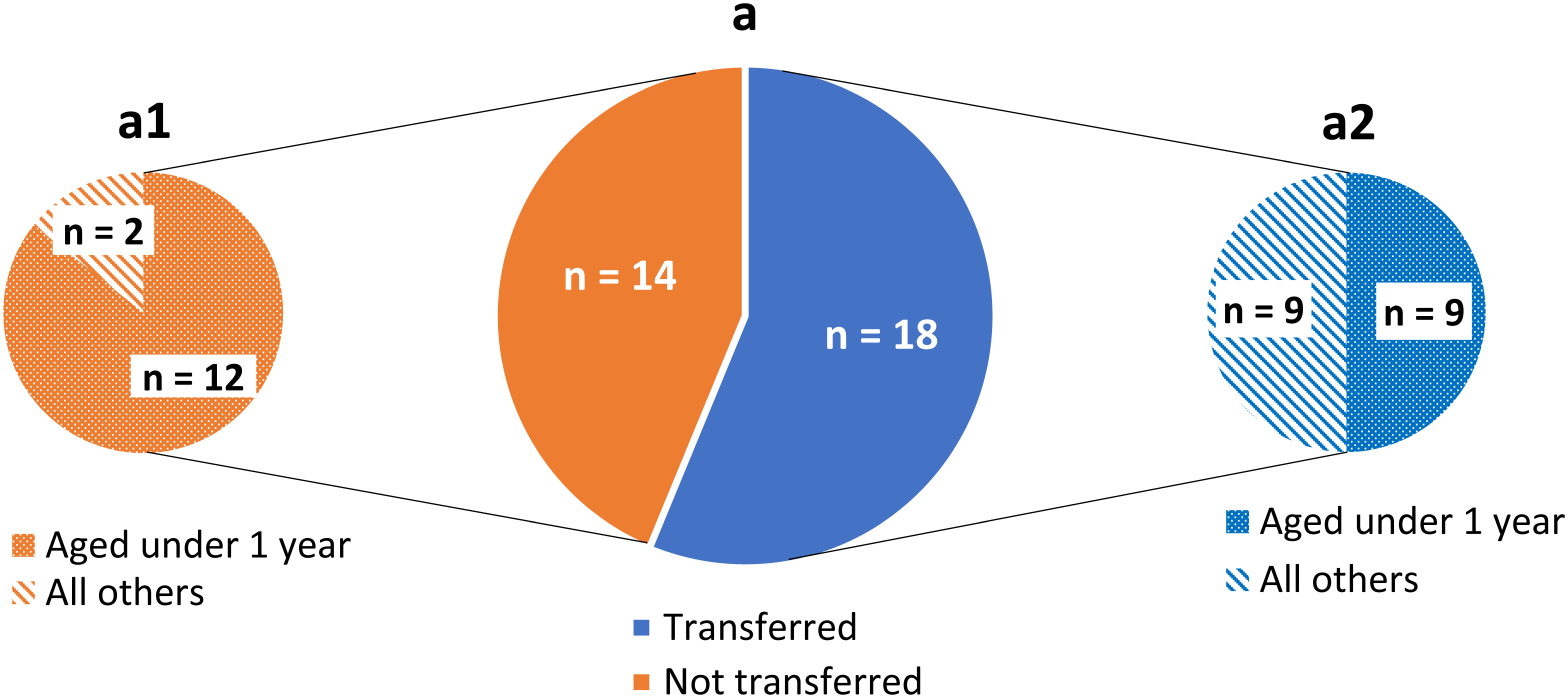
Proportion of patients transitioning to HPN according to age. (a) Proportion of patients transitioning to HPN; (a1) proportion of patients aged under 1 year who did not transition to HPN; (a2) proportion of patients aged under 1 year who transitioned to HPN.

Of the 32 patients starting the index PN episode with PN at the hospital, 18 transferred to HPN during that episode (although some patients subsequently returned to PN at the hospital; Fig. 5). This means that the maximum number of patients who received HPN at any point in the study was 34 (70.8% of all patients).

Patients aged under 1 year were more likely not to be transferred to HPN. This group accounted for 50.0% of patients (9/18) who were transferred and 85.7% (12/14) of those who were not transferred (Fig. 5).

Compared with patients who were not transferred, there was a slightly higher proportion of certain comorbidities among patients who were transferred, namely: comorbid liver disease (35.7% vs. 44.4%), malnutrition (7.1% vs. 38.9%), and dehydration (7.1% vs. 16.7%). The numbers of patients with other comorbidities were too small for meaningful comparisons. Rates of transfer to HPN among patients who underwent different surgeries were similar.

In general, complications of PN at the hospital were more likely to prevent the transfer to HPN. Notable exceptions were mechanical complications and CRBSIs respectively, found in 33.3% and 77.8% of the 18 patients transferred to HPN compared with 0.0% and 71.4% of the 14 patients who were not transferred. No patient who transferred to HPN experienced refeeding syndrome, pulmonary embolism, phlebitis/thrombophlebitis, ischemic stroke, or transient ischemic attack.

#### Patient care

The mean (SD) duration of hospitalization for the 30 patients receiving PN at the hospital at index was 395.5 (466.8) days. These patients experienced an average of 10.2 recurrent hospitalizations. Most of these patients (83.3%) were discharged home. ADL scores at the time of discharge were available for 12 (40%) patients; for all the ADLs assessed, at least 8 (two‐thirds) out of the 12 patients showed total dependence.

Two children (6.7%) died during the study period: both were newborns with neonatal necrotizing enterocolitis and neither had undergone surgery. These patients had received PN for an average of 9.7 months before death. Complications related to hospital PN in these patients included CRBSI, sepsis, bacteremia (one patient each), venous thromboembolism (one patient), and IFALD (both patients). Both children received antibiotics, anticoagulants, and gastric antisecretory agents. One patient also received antidiarrheals.

## Discussion

This study provides a comprehensive real‐world picture of the causes and treatment patterns in PN‐dependent pediatric patients with SBS‐IF in Japan.

Children with PN episodes account for around 10% of all patients with PN episodes in the MDV database. The majority were aged under 6 years with an index event of PN at the hospital and were smaller (shorter and lighter) than the national average for Japanese children of the same age. Mean standardized weights are 3.0–6.3 kg (0–3 months), 5.9–9.4 kg (3–12 months), and 8.7–19.0 kg (1–5 years).^14^ Mean standardized heights are 49–62 cm (0–3 months), 60–75 cm (3–12 months), and 74–113 cm (1–5 years).^14^

The patients included in this study had a history of mechanical bowel obstruction (25.0%), functional bowel disorder (21.0%), Hirschsprung's disease (21.0%), or surgery for small bowel obstruction (17.0%). Hirschsprung's disease is a rare congenital condition characterized by the absence of neurons (aganglionosis) in a portion of the intestinal tract, usually the distal colon, leading to bowel obstruction ultimately.^15^ This is consistent with the findings of a 2016 study of 109 infants with SBS, which showed that the leading causes of SBS were bowel obstruction: necrotizing enterocolitis (NEC; 34.0%), gastroschisis (20.0%), midgut volvulus (14.0%), intestinal atresia (10.0%), and Hirschsprung's disease (9.0%).^16^

In this study, fewer than half of patients (45.8%) were weaned from PN; this is somewhat lower than in a study by Sigalet *et al*. (duration: 8 years; mean follow up 82 months)^17^ but similar to that found by Sparks *et al*. (duration of study: 12 years; median follow up 52 months).^16^ More than half of the patients that were weaned (54.5%) were aged under 1 year. In children of this age, the residual intestinal tract can be expected to grow as the child grows: this is advantageous for adaptation and weaning from PN at hospital. On average, children who were weaned received PN for 15 months before weaning. The difference between index PN duration and time to first weaning was 39.6 days (i.e., the weaning process took about 6 weeks).

The most frequent complications of PN in this cohort were infections (79.0%) and IFALD (65.0%). The high incidence of sepsis in this study (79.0%) is in agreement with the incidence of sepsis in a 2017 Chinese study (85.0%)^18^ and the frequency of IFALD is also as expected.^3^, ^4^, ^11^ It is known that pediatric patients receiving long‐term PN are at higher risk of IFALD as seen by the higher frequency of cholestasis and rapid progression to severe hepatic fibrosis, cirrhosis, and end‐stage liver failure.^3^, ^5^

Parenteral nutrition and its associated complications can have an immediate impact on the patients' quality of life and have long‐term impacts on the children's growth and nutritional status even after weaning to a normal diet.^11^, ^19^, ^20^ The patients in this study endured many hospitalizations; even at home, two‐thirds of patients needed help with ADL. Children with SBS, therefore, require a comprehensive treatment strategy and careful monitoring involving a multidisciplinary team of physicians and other specialists to minimize the time they are dependent on PN.^5^, ^19^, ^21^

Home parenteral nutrition has been described as a lifeline for normalcy in living with a better quality of life for people with SBS.^19^, ^22^ The transition from PN at hospital to HPN is therefore an important goal.

In this study, 56.0% of the patients with an index event of PN at hospital were transferred to HPN (although some subsequently reverted to PN at hospital). This is lower than expected from a recent review, which found that around 70.0% of children transition from PN at hospital to HPN.^3^ In this study, the majority of patients who did not transition to HPN were under 1 year old. These results may reflect the lack of a system to support transition to HPN in Japan, especially for infants. Pediatric HPN has a higher frequency of liver damage and sepsis than adult HPN,^3^, ^23^ so multidisciplinary support is crucial for a safe transition to HPN.^24^, ^25^, ^26^

Mortality rates were examined in an *ad hoc* analysis in this study. Two patients (7.0%) died during the follow‐up period; this is lower than expected from earlier studies, which found mortality rates of more than 10.0%.^5^ However, those studies also had much longer follow‐up periods.

There are some limitations to this study. First, as the information was collected from the database, certain data such as the length of the remaining small intestine and variables relating to clinical test results were not available for analysis. Second, the results of this study may not be generalizable to all pediatric patients with SBS‐IF in Japan because the database included hospitals with ≥200 beds, (approximately 30.0% of all hospitals in Japan); thus small hospitals and clinics were underrepresented. Third, a small number of pediatric patients was included in this study, even though the reported incidence of SBS in newborns is 24.5 per 100 000 live births.^27^ One of the reasons for the small sample size may be because the database does not cover all the acute care children's hospitals at which most pediatric patients are initially treated. Another reason may be that most pediatric patients were not included in this study as the requirement was continuous PN for ≥6 months. In this study, a cut‐off of ≥6 months was considered to ensure the inclusion of SBS cases with no pathological change and patients who had become PN‐dependent after a period of stability. Conversely, some patients may have been included twice if they had been transferred to another hospital during PN as they would be allocated a new patient ID and would not be traceable using the original patient ID. Other patients may have been omitted if they were not identified by the disease receipt code for SBS (8841646). To mitigate this risk, we defined the study population by combining evidence of PN therapy and evidence of underlying diseases that can lead to SBS.

### Conclusions

We believe that this is the first study to provide a comprehensive overview of the causes of SBS in Japanese pediatric patients and how they respond to treatment. Only a minority of patients were weaned from PN, and only around half of them transitioned from PN at hospital to HPN, which might offer them a greater quality of life. The frequency and severity of complications arising from prolonged PN provide an impetus for finding ways to improve this situation.

It is also hoped that the outcomes of this analysis may be useful in the development of guidelines for rehabilitation programs, as well as supporting decision making for SBS diagnosis and treatment.

## Disclosures

This work was supported by Takeda Pharmaceutical Company Ltd. Tsunekazu Mizushima received honoraria and consulting fees from Takeda Pharmaceutical Company Ltd., grants or funds from Takeda Pharmaceutical Company Ltd., Taiho Pharamaceutical Co. Ltd., Chugai Pharamaceutical Co. Ltd., Yakult Honsha Co. Ltd., Eli Lilly Japan K.K., Sanofi K.K., Mitsubishi Tanabe Pharma Corporation, Kaken, Astellas Pharma Inc., Daiichi Sankyo Co. Ltd, and MSD K.K. The Department of Therapeutics for Inflammatory Bowel Diseases is supported by an unrestricted grant from Kinshukai Medical Corporation. Shiro Nakamura received honoraria from Takeda Pharmaceutical Company Ltd., AbbVie Inc, Mitsubishi Tanabe Pharma Corporation, EA Pharma Co. Ltd., Mochida Pharmaceutical Co. Ltd., and Janssen Pharmaceutical Co. Ltd., grants or funds from Mitsubishi Tanabe Pharma Corporation, Mochida Pharmaceutical Co. Ltd., EA Pharma Co. Ltd., Kyorin Holdings Inc, AbbVie Inc, JIMRO Co. Ltd., Zeria Pharmaceutical Co. Ltd. Eri Udagawa is an employee of Takeda Pharmaceutical Company Ltd. Hiroomi Okuyama received honoraria and consulting fees from Takeda Pharmaceutical Company Ltd. Jovelle Fernandez is an employee of Takeda Pharmaceutical Company Ltd., and owns restricted stocks in Takeda and GlaxoSmithKline. Yuko Tazuke has no conflict of interest.

## Author contributions

Y.T., E.U., T.M., S.N., J.F., and H.O.: designed the study, provided data analysis and interpretation, critical revision, and approved the manuscript and agreed on accountability for all aspects of the work. All authors read and approved the final manuscript.
